# Zika and Dengue Interactions in the Context of a Large Dengue Vaccine Clinical Trial in Latin America

**DOI:** 10.4269/ajtmh.20-0635

**Published:** 2020-11-09

**Authors:** Betzana Zambrano, Fernando Noriega, Gustavo H. Dayan, Doris Maribel Rivera, José Luis Arredondo, Humberto Reynales, Kleber Luz, Carmen Deseda, Matthew I. Bonaparte, Edith Langevin, Yukun Wu, Margarita Cortés, Stephen Savarino, Carlos A. DiazGranados

**Affiliations:** 1 Clinical Sciences Department, Sanofi Pasteur, Montevideo, Uruguay;; 2 Clinical Sciences Department, Sanofi Pasteur, Swiftwater, Pennsylvania;; 3 Inversiones en Investigación Médica, Pediatric Infectious Diseases Research, Tegucigalpa, Honduras;; 4 Unidad de Investigación Clínica, Clinical Research Department, Instituto Nacional de Pediatría, Mexico City, Mexico;; 5 Clinical Research Department, Centro de Atención e Investigación Médica (CAIMED), Bogotá, Colombia;; 6 Universidade Federal do Rio Grande do Norte, Natal, Brazil;; 7 Caribbean Travel Medicine Clinic, San Juan, Puerto Rico;; 8 Translational Sciences and Biomarkers Department, Sanofi Pasteur, Swiftwater, Pennsylvania;; 9 Sanofi, Health Economics and Value Access, Lyon, France;; 10 Global Health Department, Sanofi, Bogota, Colombia

## Abstract

A phase III dengue vaccine trial including 9- to 16-year-olds in Latin America (NCT01374516) was ongoing at the time of a Zika outbreak. We explored interactions between dengue and Zika, in the context of dengue vaccination. Symptomatic virologically confirmed Zika (VCZ) was evaluated using acute-phase sera from febrile participants (January 2013–March 2018). Neutralizing antibody geometric mean titers (GMTs) were evaluated pre- and post-Zika outbreak (months 25 and 72) in 2,000 randomly selected participants. Baseline dengue serostatus was determined using the plaque reduction neutralization test or inferred post hoc using nonstructural protein 1 IgG ELISA at M13 (case–cohort analysis). Vaccine efficacy against VCZ and serologically suspected Zika (SSZ) was estimated. Overall, 239/10,157 (2.4%) acute-phase samples were VCZ positive during the study. Dengue vaccine efficacy against VCZ was 27.8% (95% CI: 0.3; 47.7) among baseline dengue-seropositive participants. No vaccine effect was evident against SSZ. Zika antibody GMTs increased from pre- to post-Zika epidemic, with smaller increases observed for participants who were dengue seropositive at baseline than for those who were dengue seronegative: post-/pre-Zika GMT ratios for baseline dengue-seropositive participants were 21.5 (vaccine group) and 30.8 (placebo); and for dengue seronegatives, 88.1 and 89.5, respectively. Dengue antibody GMTs post-Zika were higher in dengue vaccine and placebo recipients with SSZ than those without SSZ in both dengue seropositives and seronegatives. Dengue vaccine did not enhance symptomatic Zika illness in dengue-seropositive individuals, rather it reduced the risk of VCZ. Zika infection boosted preexisting vaccine-induced or naturally occurring dengue-neutralizing antibodies.

## INTRODUCTION

Latin America is a region of high endemicity for the mosquito-borne flavivirus dengue, with considerable activity reported over the last 30 years.^1,2^ In 2016, more than 2.38 million dengue cases were reported, of which 1.5 million cases occurred in Brazil alone.^3^ An unprecedented outbreak of Zika, another mosquito-borne flavivirus, associated with clusters of microcephaly in newborns, Guillain–Barré syndrome, and other neurological disorders, was declared a public health emergency of international concern in February 2016 by the WHO.^4,5^ The first confirmed cases of Zika infection were reported in Brazil in March 2015, and by March 2016, 33 countries and territories were affected.^6–8^

The flavivirus envelope (E) glycoprotein, a major target for neutralizing antibody responses, is structurally similar between dengue and Zika viruses (ZIKV), with up to 51% amino acid identity within E protein domain II.^9–11^ As such, dengue-neutralizing antibodies may be cross-protective against Zika. Alternatively, preexisting antibodies against one of the flaviviruses may enhance disease upon infection with the other. Although studies in vitro and in mice models suggest that Zika infection is enhanced in the presence of dengue antibodies,^10–12^ this has not been demonstrated in vivo.^13–16^ On the contrary, antibodies to dengue virus (DENV) have been shown to offer protection against Zika infection in rhesus monkeys,^17^ and human epidemiological studies suggest that dengue antibodies following “natural” dengue infection can be protective against Zika.^18,19^ A recent in vitro study using serum samples from patients with dengue fever or dengue hemorrhagic fever demonstrated both neutralizing and enhancing effects of anti-dengue antibodies on Zika infection depending on antibody concentration.^20^

Sanofi Pasteur’s phase III dengue vaccine efficacy trial (CYD15), comprising more than 20,000 participants from dengue-endemic countries in Latin America, was ongoing at the time of the first virologically confirmed Zika (VCZ) case in the outbreak in Brazil.^6^ The CYD15 study provided a unique opportunity to assess the interactions between Zika and dengue from immunological and clinical perspectives, in the context of dengue vaccination. Here, we describe the occurrence of symptomatic Zika illness and serologically suspected Zika (SSZ) infection, in all participants and according to dengue serostatus, and the potential impact of dengue vaccination. We also investigated the effects of dengue on Zika antibody responses and of Zika infection on dengue antibody responses.

## METHODS

### Study design and participants.

The study design and primary efficacy and safety outcomes of the CYD15 phase III study (NCT01374516) have been previously described.^21^ In total, 20,875 healthy children and adolescents aged 9–16 years were enrolled between June 2011 and March 2012. Participants were randomized 2:1 to receive three injections of the live, attenuated, tetravalent dengue vaccine (CYD-TDV [Dengvaxia^®^, Neuville sur Saône, France]) or 0.9% saline placebo, at months (M) 0, 6, and 12, in 22 sites across Brazil, Colombia, Honduras, Mexico, and Puerto Rico. Participants (*n* = 2000) enrolled during the first 2–4 months of the CYD15 trial were randomly assigned to an immunogenicity subset, for reactogenicity and immunogenicity assessment.

**Figure 1.**
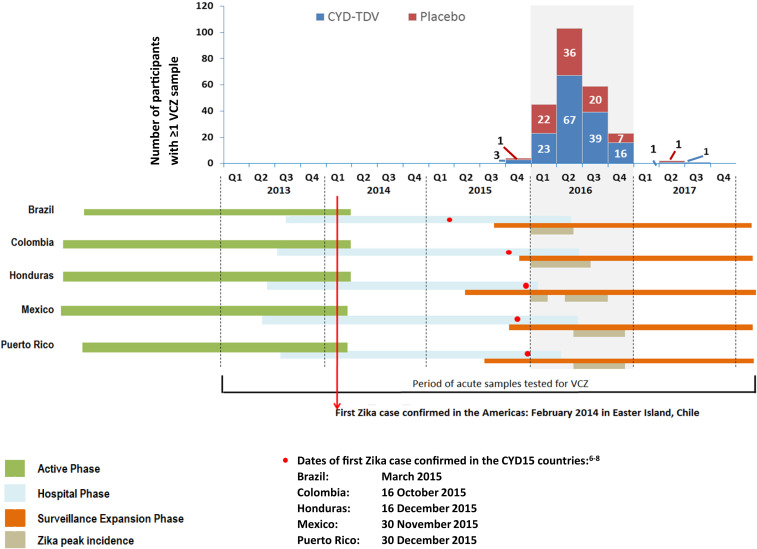
Virologically confirmed Zika (VCZ) cases per quarter, per calendar year in the live, attenuated, tetravalent dengue vaccine (CYD-TDV) and placebo groups. Participants were randomized in a 2:1 ratio to CYD-TDV and placebo groups. The colored bars below the graph depict when the different phases of the trial were established and the period of peak Zika incidence for each country. During the hospital phase, only samples from hospitalized cases with suspected dengue were tested; no VCZ cases were detected during this phase of the trial. This figure appears in color at www.ajtmh.org.

The CYD15 study duration was 6 years and comprised a 2-year active surveillance phase (M0–M25; June–August 2011) to detect symptomatic dengue, regardless of severity; followed by a hospital phase of variable duration (in some cases up to the study end in March 2018 [M72]), detecting only hospitalized dengue events; and a surveillance expansion phase (SEP) from May 2015 to the study end, for which active surveillance of both hospitalized and nonhospitalized cases was reinstated.^22^ Exact dates and duration of each study phase differed for each country depending on their national regulatory approval processes (Figure 1, Supplemental Table S1).

The CYD15 study protocol was amended after the first virological confirmation of Zika in March 2015^6^ for additional Zika testing and for the investigation of the potential interaction between dengue and ZIKVs. The study complied with the Declaration of Helsinki, good clinical practice (GCP) guidelines, and relevant local regulations. Ethics review committees and regulatory agencies approved the protocol, amendments, and consent and assent forms. No additional collection of samples was required for the additional Zika testing, but consent was required to undertake Zika testing in the samples collected in Brazil and Colombia as per local regulations.

### Assessment of virologically confirmed Zika.

Detection of VCZ was performed as a differential diagnosis for dengue infection for all acute-phase serum samples collected from the start of 2013 to the end of the study (March 2018). Febrile dengue illness was documented during the active and SEP study phases (active surveillance), as previously described,^21^ with acute-phase blood samples taken within five days after fever onset (≥ 38°C for at least two consecutive days). During the hospital phase, acute-phase samples were collected for participants admitted to hospital with acute febrile illness. A Zika-specific reverse transcriptase quantitative polymerase chain reaction (RT-qPCR; performed at ARUP laboratories, Salt Lake City, UT) was used on these acute-phase samples, with primers and probes specific for the Zika nonstructural protein 3 (NS3) gene.^23^ A positive RT-qPCR result was classified as VCZ.

Zika signs and symptoms, based on those most frequently reported by the WHO or CDC,^24,25^ were described for all participants identified with VCZ using data previously collected for the assessment of dengue signs and symptoms.^21^

### Assessment of SSZ and evaluation of Zika antibody response.

Zika-neutralizing antibody titers were evaluated in serum samples from the immunogenicity subset at two time points: M25, before the start of the Zika epidemic (pre-Zika; as indicated by the first serologically confirmed Zika cases),^6–8^ and M72, after the peak incidence of Zika (post-Zika). Zika antibody titers were measured using a Zika microneutralization test (performed by Sanofi Pasteur GCI, Swiftwater, PA).^26^ Zika seropositivity was defined as a titer ≥ 100 (1/+dil). The high cutoff titer for Zika seropositivity was selected to ensure high assay specificity (100% specificity and 98% sensitivity using a cutoff titer of 100, as previously shown in VCZ samples collected from dengue-endemic areas).^26^ Participants were considered to have SSZ if they had a titer ≥ 100 (1/dil) at M72, regardless of M25 titers.

### Evaluation of dengue antibodies and dengue baseline serostatus.

As previously described,^21,22^ dengue serotype–specific neutralizing antibodies were measured using the 50% plaque reduction neutralization test (dengue PRNT_50_) for participants of the immunogenicity subset before study injection (M0), after the second injection (M7), after the third injection (M13), and approximately yearly thereafter until the end of the study (M25, M36, M48, M60, and M72). Dengue seropositivity was defined as a titer ≥ 10 (1/dil) for at least one dengue serotype at baseline; dengue seronegativity was defined as a titer < 10 (1/dil) for all four serotypes at baseline.

For the analysis of VCZ by baseline dengue serostatus, the number of VCZ events in the immunogenicity subset was too low to obtain meaningful results. Therefore, a case–cohort framework was used, whereby baseline dengue serostatus was inferred for a randomly selected subcohort (comprising approximately 10% of the entire study population) and for all participants with a VCZ event (i.e., cases) by measurement of anti-dengue NS1 IgG by ELISA on samples taken at M13.^27^ A cutoff of ≥ 9 ELISA units/mL was selected to minimize the rates of false-seronegative results.^27^

### Statistical analyses.

The occurrence of VCZ and clinical signs and symptoms were evaluated in the safety analysis set, which included all participants who received at least one vaccine dose and who did not have serious noncompliance to GCP; participants were analyzed according to the treatment received at the first dose. The occurrence of SSZ infection was evaluated in the immunogenicity subset (by treatment received and by baseline dengue serostatus measured by PRNT_50_); Zika and serotype-specific dengue antibody responses were determined in the full analysis set for immunogenicity, defined as participants of the immunogenicity subset who received at least one injection, who did not have serious noncompliance to GCP, and who had a result available from a blood sample drawn after receipt of study injection(s).

RR for the occurrence of VCZ in the CYD-TDV versus the placebo group was calculated in the overall study population, based on the number of VCZ cases (individual participants may have had one or more episodes of Zika) and the cumulative person-years followed, as described previously for dengue^21^; 95% CIs for vaccine efficacy (VE) were calculated using the exact method (Breslow and Day).^28^

Hazard ratios (HRs) by dengue serostatus (seropositive or seronegative) were estimated in the post hoc case–cohort analysis of VCZ using a weighted Cox proportional-hazards regression,^27^ with Wald 95% CI computed. VE against VCZ was determined from the HR estimate, such that VE = (1 − HR) × 100 in the selected subcohort.

The frequency of Zika clinical signs and symptoms was evaluated descriptively. Data were presented as total numbers of cases, means, and two-sided 95% CIs or the number and percentage of VCZ cases with specified clinical signs and symptoms. Results were stratified by treatment group (CYD-TDV or placebo) and overall dengue serostatus.

Antibody geometric mean titers (GMTs) were calculated using the log_10_ transformed individual titers with 95% CI estimated assuming that these were normally distributed. Antilog transformations were applied to provide GMTs and 95% CIs on their original scale. GMTs were reported overall, by baseline dengue serostatus and by treatment group.

## RESULTS

### Occurrence of VCZ cases.

From January 2013 to March 2018, 10,157 acute samples from 5,321 participants were tested for Zika virus. In Brazil and Colombia, samples were tested for participants who provided consent (844 [25.3%] participants in Brazil and two [0.02%] in Colombia did not provide consent). Overall, 239/10,157 (2.4%) samples from 237 participants were VCZ: 87/3,595 in the placebo group and 152/6,562 in the CYD-TDV group (Supplemental Table S2). All VCZ episodes were detected during the SEP (active surveillance), during which there was overlap with the peak in Zika incidence in each of the study countries according to epidemiological records (Figure 1, Supplemental Table S1). Brazil had one of the lowest numbers of acute samples available for testing for Zika and the lowest proportion of samples testing positive for VCZ (2/1,017; 0.2% of samples). Mexico had the highest proportion of VCZ detected (63/1,053; 6.0% of samples) (Supplemental Table S2). Overall, the RR for the occurrence of VCZ in the CYD-TDV versus the placebo group was 0.86 (95% CI: 0.65; 1.13), with the lowest RR in Colombia and the highest in Puerto Rico (not calculated in Brazil because of too few cases). Notably, the CIs included the null value of 1 in each country (Table 1).

**Table 1:** Incidence of VCZ cases from the start of 2013 by country—safety analysis set

	CYD-TDV		Placebo		RR
	Cases/person-years	Annual incidence rate (95% CI)	Cases/person-years	Annual incidence rate (95% CI)	
All countries	150/59,744	0.3 (0.2; 0.3)	87/29,740	0.3 (0.2; 0.4)	0.86 (0.65; 1.13)
Brazil	2/10,371	< 0.1 (0.0; 0.1)	0/5,144	0.0 (0.0; 0.1)	NC
Colombia	29/27,847	0.1 (0.1; 0.1)	21/13,895	0.2 (0.1; 0.2)	0.69 (0.38; 1.27)
Honduras	70/7,868	0.9 (0.7; 1.1)	45/3,894	1.2 (0.8; 1.5)	0.77 (0.52; 1.15)
Mexico	43/10,333	0.4 (0.3; 0.6)	19/5,140	0.4 (0.2; 0.6)	1.13 (0.64; 2.05)
Puerto Rico	6/3,326	0.2 (0.1; 0.4)	2/1,668	0.1 (0.0; 0.4)	1.50 (0.27; 15.24)

CYD-TDV = live, attenuated, tetravalent dengue vaccine; NC = not calculated; VCZ = virologically confirmed Zika. Person-years, number of person-years followed during the study period; cases, number of participants with at least one VCZ episode. The annual incidence rate was calculated based on cases among the number of person-years × 100; RR was based on the ratio of annual incidence.

**Figure 2.**
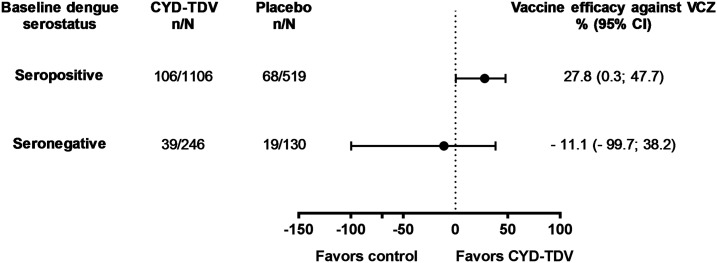
Efficacy of the live, attenuated, tetravalent dengue vaccine (CYD-TDV) against virologically confirmed Zika (VCZ) by dengue baseline serostatus (as per nonstructural protein 1 [NS1] titers at M13) throughout the study period, from 2013 – case–cohort analysis. Participants with virologically confirmed dengue before month 13 or with undetermined dengue serostatus at M13 were excluded from analysis. Baseline dengue status was determined by NS1 at month 13 using a threshold of 9 EU/mL. VE was inferred from the proportional hazard ratio: VE = (1 − HR) × 100. N = the total number of participants included in the subcohort with serostatus and treatment group as specified; n = number of participants with virologically confirmed Zika (cases), with available data for the relevant endpoint; VCZ = virologically confirmed Zika.

In a *post hoc* case–cohort analysis, VCZ was detected in 174 participants who were dengue seropositive at baseline (inferred from NS1 assay at M13); the estimated VE for CYD-TDV against VCZ in these participants was 27.8% (95% CI: 0.3; 47.7) (Figure 2). Among those dengue seronegative at baseline, 58 had VCZ, with an estimated CYD-TDV efficacy against VCZ of −11.1% (95% CI: −99.7; 38.2) (Figure 2). Within this case–cohort framework, VCZ cases occurred with similar frequency between baseline dengue-seropositive and -seronegative individuals in the placebo group, but in the CYD-TDV group, VCZ occurred less frequently in dengue-seropositive participants than -seronegative participants (Figure 2).

### Clinical manifestations of VCZ episodes.

**Figure 3.**
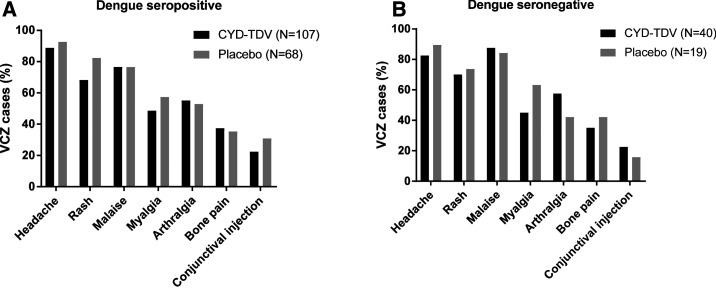
Clinical signs and symptoms reported for virologically confirmed Zika (VCZ) cases occurring throughout the study, by baseline dengue serostatus (safety analysis set). The *y*-axis shows the percentage of virologically confirmed Zika cases with available data for the relevant end point, who fulfilled criteria for the specified sign or symptom. Baseline dengue serostatus was inferred from dengue nonstructural protein 1 assay at M13. N = the number of virologically-confirmed Zika cases with baseline dengue serostatus as indicated; VCZ = virologically-confirmed Zika.

Regardless of dengue serostatus at baseline, the most common clinical signs and symptoms for VCZ cases in the CYD-TDV and placebo groups were headache, malaise, and rash (Table 2). Among participants with VCZ who were dengue seropositive, CYD-TDV recipients tended to report rash, myalgia, and conjunctival injection less frequently than the placebo group (Figure 3A). Among those who were dengue seronegative, there was a trend toward less frequent reporting of symptoms in the CYD-TDV group, except for malaise, arthralgia, and conjunctival injection (Figure 3B).

**Table 2:** Clinical signs and symptoms in VCZ cases—safety analysis set

Sign or symptom	CYD-TDV (*N* = 152)	Placebo (*N* = 87)
Number of days of fever, mean (95% CI)	3.7 (3.26; 4.12)	3.2 (2.81; 3.67)
Number of days of clinical symptoms, mean (95% CI)	8.6 (6.77; 10.40)*	7.9 (7.12; 8.58)
Symptom, *n* (%)		
Headache	133 (87.5)	80 (92.0)
Rash	103 (67.8)	70 (80.5)
Malaise	120 (78.9)	68 (78.2)
Myalgia	73 (48.0)	51 (58.6)
Arthralgia	85 (55.9)	44 (50.6)
Bone pain	54 (35.5)	32 (36.8)
Conjunctival injection	33 (21.7)	24 (27.6)

CYD-TDV = live, attenuated, tetravalent dengue vaccine; n (%) = number and percentage of cases with available data fulfilling specified item; *N* = number of VCZ cases from 2013 included in the analysis; VCZ = virologically confirmed Zika. The signs and symptoms presented are considered are the most frequently reported for Zika according to the WHO and CDC.^24,25^

* Number of participants included for this end point, *N* = 151.

### Occurrence of SSZ (irrespective of symptoms).

Serologically suspected Zika was detected in 694/1,539 (45.1%) participants who had a sample at M72: 180/227 (79.3%) in Honduras, 339/712 (47.6%) in Colombia, 103/269 (38.3%) in Mexico, 23/92 (25.0%) in Puerto Rico, and 49/239 (20.5%) in Brazil.

**Figure 4.**
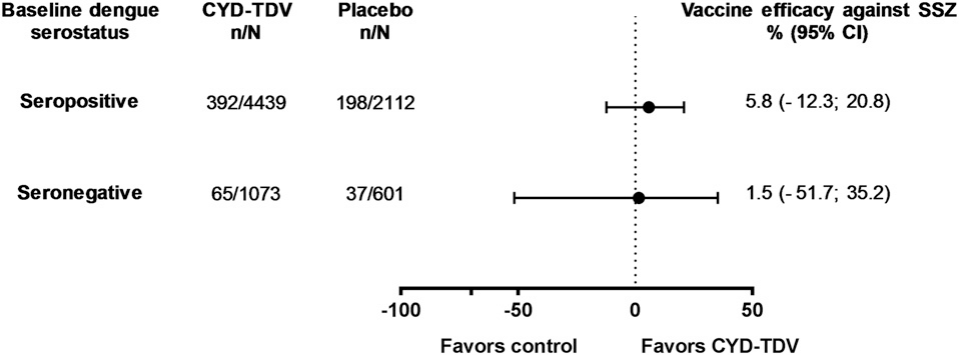
Efficacy of the live, attenuated, tetravalent dengue vaccine (CYD-TDV) against serologically suspected Zika (SSZ) from the start of 2013 to the study end by baseline dengue serostatus (50% plaque reduction neutralization test [PRNT_50_] at M0)—immunogenicity subset. *N*, number of person-years followed from the start of 2013; *n*: number of participants with Zika titers (MN) at M72 ≥ 100 1/dil; SSZ, serologically suspected Zika. Baseline dengue status by PRNT_50_ at M0.

CYD–TDV VE estimates against SSZ were 5.8 (95% CI: −12.3; 20.8) in dengue-seropositive participants and 1.5 (95% CI: −51.7; 35.2) in those who were seronegative (Figure 4). SSZ was documented more frequently in individuals who were dengue seropositive at baseline than in those who were seronegative in both CYD-TDV and placebo groups.

### Zika antibody responses.

**Figure 5.**
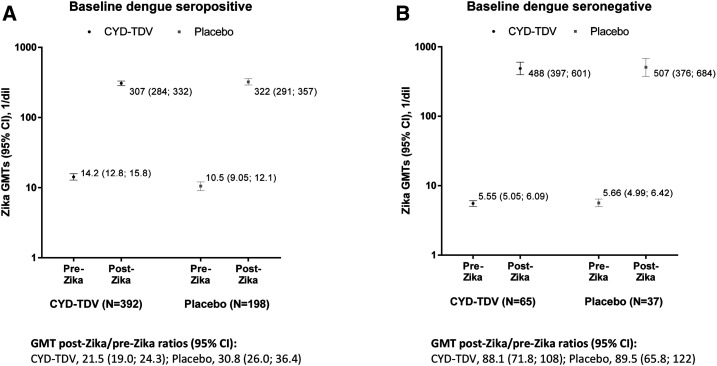
Zika geometric mean titers (GMTs) before and after the Zika epidemic, by baseline dengue serostatus and by treatment group—immunogenicity subset. Zika antibody GMTs, measured by Zika microneutralization assay, with 95% CI, are plotted on the graphs, with data labels showing point estimates. Only participants with Zika titers (MN) at M72 ≥ 100 1/dil were included in this analysis. GMT, geometric mean titer; Pre-Zika, study month 25, before the first serologically confirmed Zika cases reported by national surveillance systems; Post-Zika, study month 72, after the peak incidence of observed Zika. Baseline dengue seronegative participants are defined as those with dengue 50% plaque reduction neutralization test (PRNT_50_) titers < 10 (1/dil) against all four serotypes at baseline; baseline dengue-seropositive participants are defined as those with dengue PRNT_50_ titers ≥ 10 (1/dil) against at least one dengue serotype at baseline.

Zika antibody GMTs at the pre–Zika-epidemic time point were below the defined threshold for Zika seropositivity in both CYD-TDV and placebo groups but were higher for participants who were dengue seropositive at baseline than those who were seronegative (Figure 5). Zika GMTs increased from pre- to post-Zika time points, with a smaller relative increase among baseline dengue-seropositive participants than seronegative participants in both study groups; GMT ratios post-/pre-Zika for baseline dengue-seropositive participants were 21.5 (19.0; 24.3) and 30.8 (26.0; 36.4) in the CYD-TDV and placebo groups, respectively, and for baseline dengue-seronegative participants, 88.1 (71.8; 108) and 89.5 (65.8; 122) for the two study groups, respectively. Zika antibody GMTs at the post-Zika time point were similar between CYD-TDV and placebo groups for both baseline dengue-seropositive and -seronegative participants (Figure 5).

### Dengue-neutralizing antibody titers pre- and post-Zika.

**Figure 6.**
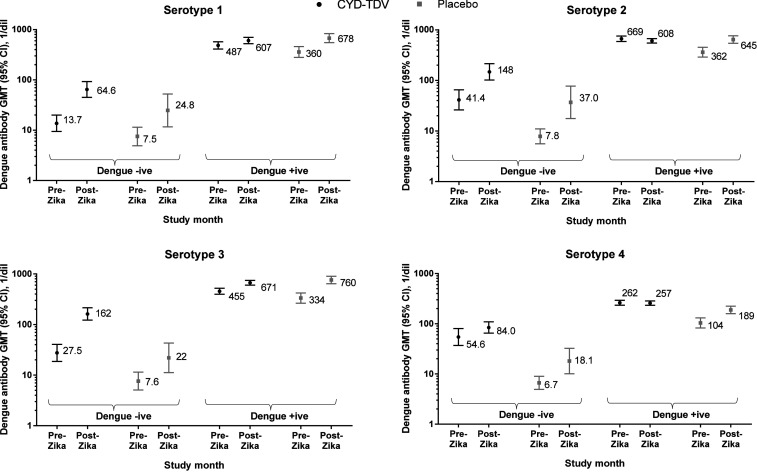
Dengue serotype–specific antibody responses before and after peak Zika incidence in those with (**A**) and without (**B**) serological evidence of Zika by baseline dengue serostatus in the immunogenicity subset. Pre-Zika represents study month 25, before the first serologically confirmed Zika cases reported by national surveillance systems. Post-Zika represents study month 72, after the peak incidence of observed Zika. Participants with serological evidence of Zika had Zika titers (microneutralization assay) at M72 ≥ 100 1/dil; participants without serological evidence of Zika had M72 titers < 100 1/dil. Dengue baseline serostatus was determined by 50% plaque reduction neutralization test at M0; dengue baseline seronegative (dengue negative) was defined as titers < 10 (l/dil) against all four serotypes at baseline and dengue baseline seropositive (dengue positive), titers ≥ 10 (l/dil) against at least one dengue serotype at baseline. CI = confidence interval; GMT = geometric mean titer; M = month.

Among participants who had serological evidence of Zika at M72 and who were dengue seropositive at baseline, dengue GMTs increased from the pre-Zika to post-Zika time points for all serotypes in the placebo group (Figure 6A). Dengue GMTs increased to a lesser extent for serotypes 1 and 3 in the CYD-TDV group, with no trend toward an increase observed for serotypes 2 and 4, such that dengue antibody titers reached similar levels post-Zika (overlapping 95% CIs) in placebo and CYD-TDV recipients for all serotypes, except serotype 4; GMTs against serotype 4 remained significantly higher in the CYD-TDV group than in the placebo group (Figure 6A). Among participants who had serological evidence of Zika at M72 and who were dengue seronegative at baseline, dengue antibody GMTs increased from pre- to post-Zika time points for each serotype, in both treatment groups, with GMTs remaining lower in the placebo groups than in the CYD-TDV group for all serotypes (Figure 6A).

Among participants without serological evidence of Zika at M72, dengue antibody GMTs were similar or decreased from pre- to post-Zika in CYD-TDV recipients, for both baseline dengue-seropositive and -seronegative participants and in placebo recipients who were dengue seropositive at baseline. Dengue GMTs showed a tendency to increase over time in placebo recipients who were dengue seronegative at baseline (Figure 6B, Supplemental Table S3).

## DISCUSSION

Our findings indicate that the Zika epidemic affected a large proportion of the CYD15 study population. This is not surprising, given that Zika and DENVs are both transmitted to humans via the same vector, the *Aedes* mosquito. We observed differences in the occurrence of Zika infection between the dengue-endemic countries included in CYD15, with VCZ reported most frequently in Mexico and Honduras and only two cases in Brazil. Similarly, rates of SSZ were highest in Honduras, followed by Colombia and Mexico, and lowest in Puerto Rico and Brazil. Other studies have reported high attack rates in Honduras and certain areas in Brazil.^18,29^ The fact that permission was not provided to test for VCZ in 25% acute-phase samples from participants in Brazil may partly explain the lower frequency of Zika infection as evidenced by either VCZ or SSZ in the Brazilian cohort in our study than other studies. Brazil was also one of the last countries to reinitiate the active surveillance phase of the study. Although the hospital phase was still ongoing at the time of the Zika outbreak in this country, mild Zika cases may not have been captured by the study surveillance. Other factors may have affected the infection rates, including differences in climatic factors, mosquito densities, and socioeconomic factors.^29^

In our study, the frequency of SSZ at M72 was higher among participants who were dengue seropositive at baseline than among those who were seronegative at baseline. This could reflect an increased risk of Zika infection in those with preexisting dengue antibodies, theoretically driven by antibody-dependent enhancement (ADE), as observed in vitro^10,12^ and in mouse models.^11^ However, enhanced ZIKV pathology due to preexisting DENV antibodies via ADE has not been observed in humans.^30^ Moreover, given the commonality in vector and transmission patterns between dengue and ZIKVs, there may be increased exposure to Zika among those exposed to dengue. However, unlike our observations for SSZ, we observed a similar frequency of symptomatic Zika, regardless of dengue serostatus at baseline among unvaccinated individuals.

Other recent studies assessing potential Zika and dengue interactions in Latin America have shown conflicting results in terms of the effect of preexisting dengue on the risk of Zika infection, ranging from a protective effect^18^ to no effect.^19,31^ Notably, Rodriguez-Barraquer et al.^18^ observed an increased frequency of Zika infection in a cohort of residents in Salvador, Brazil, with increased titers of a specific subclass of dengue NS1 antibodies (IgG3), thought to reflect recent dengue infection, whereas Gordon et al.^19^ found that prior or recent DENV immunity did not affect the overall rate of Zika infections in children in Nicaragua. However, these previous studies suggest that dengue antibodies may play a role in the modulation of symptomatic Zika disease, with dengue antibodies associated with a reduced risk of symptomatic Zika illness.^18,19^ In addition, in a recent case–control study in Brazil, prior infection with more than one dengue serotype among mothers was protective against congenital Zika syndrome in neonates.^13^

In the CYD15 study, participants were randomly assigned to receive CYD-TDV or placebo. As such, we were able to evaluate the potential effect of dengue vaccination on Zika infection and disease, with a low risk of bias. Our findings suggest that the dengue vaccine has no major effect on SSZ. In addition, CYD-TDV may reduce the risk of VCZ among individuals who were dengue seropositive at baseline; no effect of vaccination was observed for those who were dengue seronegative. The apparent protection against symptomatic Zika illness conferred by the dengue vaccine was unlikely to be mediated by Zika-specific neutralizing antibodies. However, the immune responses elicited by the dengue vaccine may have modulated the Zika immune responses. This is partially supported by the less pronounced increase in Zika antibody titers from pre- to post-Zika for vaccine recipients than placebo recipients among baseline dengue-seropositive participants. It should also be noted, however, that the less pronounced increase in Zika antibody GMTs from the pre- to post-Zika time period observed in baseline dengue-seropositive placebo recipients than baseline dengue-seronegative CYD-TDV recipients could reflect a higher magnitude of modulation of the Zika antibody response by antibodies induced by natural dengue infection than by vaccine-induced antibodies. However, there were too few cases for a meaningful analysis, and this would require further investigation. The lower frequency of some of the symptoms of VCZ observed for vaccine recipients than placebo recipients, among those who were baseline dengue seropositive, further supports the potential impact of dengue vaccination on Zika disease modulation. A potential role for the immune responses elicited by CYD-TDV in the protection against Zika disease is consistent with previous findings suggesting protection against Zika by preexisting dengue antibodies elicited by dengue natural infections.^13,18,19^

Our study also provided insights into the potential influence of Zika on dengue antibody responses. First, in placebo recipients who were dengue seropositive at baseline, dengue GMTs were higher after the Zika epidemic in those with serological evidence of Zika than in those without, indicating that Zika infection is associated with boosting of preexisting dengue-neutralizing antibodies. This may at least partly explain why the Zika epidemic was associated with a coincident decrease in dengue across the Americas.^32^ Second, among dengue-seronegative individuals, dengue antibody titers at the post-Zika time point were higher in the CYD-TDV group than in the placebo group. Thus, CYD-TDV–induced dengue antibodies may also be boosted by Zika infection. Third, the boosting of dengue antibodies by Zika infection in baseline dengue-seropositive participants was more pronounced in placebo than CYD-TDV recipients, such that the levels of dengue antibody titers in placebo recipients appeared to be similar to those in CYD-TDV vaccine recipients for all dengue serotypes at the end of study (M72), with the exception of serotype 4.

Given that CYD-TDV and placebo injections were administered a few years before the emergence of Zika in the Americas, this study cannot address how prior Zika infection may influence immune responses elicited by CYD-TDV. Previous exposure to other non-dengue flaviviruses has been suggested to exert priming effects on the CYD-TDV antibody responses.^33,34^ Of note, the Zika antibody titers that were observed in samples collected pre–Zika epidemic may reflect cross-reactivity with DENVs in individuals who may have had asymptomatic natural dengue infection (in both CYD-TDV and placebo groups), as described by others previously.^35^ However, the use of a high cutoff for the Zika assay may have helped mitigate the risk of cross-reactivity with dengue antibodies to some extent.^26^

Our study has certain limitations. Stratification by dengue serostatus was based on dengue antibodies measured approximately 2–4 years before the Zika epidemic. Therefore, our estimates cannot be interpreted as assuming with certainty lack of dengue exposure before Zika infection at the individual level in baseline dengue-seronegative subjects. It should also be noted that detection of VCZ was undertaken using acute-phase serum samples that were collected for suspected dengue infection, which included fever as a required symptom. Therefore, some cases of asymptomatic or mild symptomatic Zika infections, for which fever was not a symptom, may have been missed in this study.

Zika was unlikely to result in hospitalization in the study population and, thus, would have been unlikely to be identified during the hospital surveillance phase of the study. Thus, the identification of symptomatic Zika following the amendment of the CYD15 protocol relied largely on the reinitiation of the active surveillance of febrile illness during the SEP. The case–cohort design and application of the dengue NS1 IgG ELISA to evaluate the potential effect of CYD-TDV on VCZ have limitations that have been previously acknowledged.^27^ In addition, the evaluation of only two time points to assess SSZ did not allow for a complete assessment over time of potential interactions between Zika- and dengue-neutralizing antibody responses.

In conclusion, the present study does not provide evidence of Zika disease enhancement by CYD-TDV vaccination; our findings suggest a possible protective effect of the vaccine on symptomatic Zika illness in individuals who are dengue seropositive, the population currently recommended for CYD-TDV vaccination.^36^ Our findings also show that Zika infection may boost dengue antibodies elicited by vaccination or natural dengue infection in both dengue-seropositive and -seronegative individuals.

## Supplemental information and tables appear

Supplemental materials
