# Supplementary material for: Zika and Dengue Interactions in the Context of a Large Dengue Vaccine Clinical Trial in Latin America
Source: Am J Trop Med Hyg. 2020 Nov 9;104(1):136–44. doi: 10.4269/ajtmh.20-0635 (PMC7790115; doi:10.4269/ajtmh.20-0635)
Supplement: Supplementary file 1 [file tpmd200635.SD1.pdf]

Supplemental Table S1. Dates of Zika epidemic and CYD15 surveillance expansion phase for each country

Country	2015 Zika epidemic (epidemiological data)		CYD15 SEP start date
	**First confirmed case of Zika**	**Dates of peak incidence**	
**Brazil**[1,2]	March 2015 (Natal)	Jan to May 2016	26 Aug 2015<br>Jul 2016 (ICF completion)
**Colombia**[3]	16 Oct 2015	Jan to Jul 2016	20 Nov 2015
**Mexico**[3]	30 Nov 2015	Jun to Nov 2016	15 Oct 2015
**Honduras**[3,4]	16 Dec 2015	Jan to Feb 2016<br>May to Sep 2016	15 May 2015
**Puerto Rico**[3]	30 Dec 2015	Jun to Nov 2016	29 Jun 2015

SEP, surveillance expansion phase

Supplemental Table S2. Occurrence of virologically-confirmed Zika episodes throughout the study period (January 2013 to March 2018) by country- Safety Analysis Set

	CYD-TDV		Placebo		All	
**CYD15 study phase**	**n/M**	**%**	**n/M**	**%**	**n/M**	**%**
**All countries**						
Active surveillance	152/6455	2.4	87/3525	2.5	239/9980	2.4
Hospital surveillance	0/107	0.0	0/70	0.0	0/177	0.0
**All**	**152/6562**	**2.3**	**87/3595**	**2.4**	**239/10157**	**2.4**
**Brazil**						
Active surveillance	2/632	0.3	0/364	0.0	2/996	0.2
Hospital surveillance	0/10	0.0	0/11	0.0	0/21	0.0
**All**	**2/642**	**0.3**	**0/375**	**0.0**	**2/1017**	**0.2**
**Colombia**						
Active surveillance	29/2508	1.2	21/1310	1.6	50/3818	1.3
Hospital surveillance	0/37	0.0	0/33	0.0	0/70	0.0
**All**	**29/2545**	**1.1**	**21/1343**	**1.6**	**50/3888**	**1.3**
**Honduras**						
Active surveillance	71/2376	3.0	45/1358	3.3	116/3734	3.1
Hospital surveillance	0/37	0.0	0/16	0.0	0/53	0.0
**All**	**71/2413**	**2.9**	**45/1374**	**3.3**	**116/3787**	**3.1**
**Mexico**						
Active surveillance	44/647	6.8	19/380	5.0	63/1027	6.1
Hospital surveillance	0/19	0.0	0/7	0.0	0/26	0.0

CYD15 study phase	CYD-TDV n/M	CYD-TDV %	Placebo n/M	Placebo %	All n/M	All %
**All**	**44/666**	**6.6**	**19/387**	**4.9**	**63/1053**	**6.0**
**Puerto Rico**						
Active surveillance	6/292	2.1	2/113	1.8	8/405	2.0
Hospital surveillance	0/4	0.0	0/3	0.0	0/7	0.0
**All**	**6/296**	**2.0**	**2/116**	**1.7**	**8/412**	**1.9**

n, number of VCZ episodes; M, number of fever episodes with start date after 01JAN2013 and with Zika RT-PCR results available on acute sample

Active surveillance, data collected during the active phase of the study or surveillance expansion phase; hospital surveillance, data collected during the hospital phase of the study.

VCZ, virologically confirmed Zika.

In Brazil and Colombia, participants' consent was needed to test their blood samples for Zika virus; consent was not provided for 844 [25.3%] participants in Brazil and two participants in Colombia, hence these samples were not assayed for Zika.

Supplemental Table S3. Dengue serotype-specific antibody responses before and after peak Zika incidence in those with and without serological evidence Zika by baseline dengue serostatus in the immunogenicity subset

Dengue virus serotype	Timepoint	CYD-TDV		Placebo	
**A. Baseline dengue seronegative ($PRNT_{50}$)**					
		**Zika +ive N=63**	**Zika-ive N=129**	**Zika +ive N=36**	**Zika-ive N=75**
**Dengue 1**	**Pre-Zika**	13.7 (9.43; 20.0)	13.3 (10.4; 17.1)	7.54 (4.92; 11.5)	6.51 (5.12; 8.29)
	**Post-Zika**	64.6 (45.0; 92.8)	15.4 (12.0; 19.7)	24.8 (11.7; 52.5)	9.88 (6.99; 14.0)
	**Post-/pre-Zika ratio**	4.71 (3.20; 6.94)	1.14 (0.883; 1.47)	3.29 (1.83; 5.94)	1.52 (1.10; 2.10)
**Dengue 2**	**Pre-Zika**	41.4 (26.2; 65.6)	44.3 (32.3; 60.7)	7.84 (5.59; 11.0)	8.62 (6.33; 11.7)
	**Post-Zika**	148 (102; 215)	23.8 (18.1; 31.4)	37.0 (17.7; 77.4)	13.3 (9.14; 19.3)
	**Post-/pre-Zika ratio**	3.58 (2.20; 5.81)	0.538 (0.405; 0.715)	4.72 (2.45; 9.09)	1.54 (1.14; 2.09)
**Dengue 3**	**Pre-Zika**	27.5 (18.7; 40.5)	31.8 (24.7; 40.9)	7.62 (5.06; 11.5)	8.57 (6.03; 12.2)
	**Post-Zika**	162 (122; 215)	29.1 (22.6; 37.3)	22.0 (11.2; 43.1)	12.3 (8.49; 17.7)
	**Post-/pre-Zika ratio**	5.88 (3.98; 8.70)	0.914 (0.714; 1.17)	2.88 (1.68; 4.96)	1.43 (1.09; 1.89)
**Dengue 4**	**Pre-Zika**	54.6 (37.0; 80.5)	45.2 (34.8; 58.7)	6.66 (4.94; 8.99)	8.37 (6.18; 11.3)
	**Post-Zika**	84.0 (65.0; 109)	32.2 (25.5; 40.6)	18.1 (10.1; 32.4)	11.0 (8.13; 14.9)
	**Post-/pre-Zika ratio**	1.54 (1.13; 2.10)	0.712 (0.570; 0.890)	2.72 (1.65; 4.48)	1.29 (1.03; 1.62)
**B. Baseline dengue seropositive ($PRNT_{50}$)**					
		**Zika +ive N=391**	**Zika-ive N=438**	**Zika +ive N=196**	**Zika-ive N=188**
**Dengue 1**	**Pre-Zika**	487 (412; 576)	639 (532; 768)	360 (281; 461)	334 (246; 452)
	**Post-Zika**	607 (526; 700)	435 (369; 513)	678 (552; 832)	317 (246; 410)
	**Post-/pre-Zika ratio**	1.24 (1.11; 1.39)	0.700 (0.632; 0.775)	1.88 (1.58; 2.25)	0.951 (0.780; 1.16)
**Dengue 2**	**Pre-Zika**	669 (591; 756)	613 (537; 700)	362 (290; 453)	309 (236; 405)
	**Post-Zika**	608 (551; 672)	362 (320; 410)	645 (543; 766)	252 (196; 324)
	**Post-/pre-Zika ratio**	0.910 (0.824; 1.01)	0.582 (0.525; 0.646)	1.78 (1.46; 2.17)	0.815 (0.666; 1.00)
**Dengue 3**	**Pre-Zika**	455 (397; 521)	567 (485; 662)	334 (264; 422)	298 (219; 404)
	**Post-Zika**	671 (602; 749)	447 (390; 513)	760 (642; 898)	318 (246; 411)
	**Post-/pre-Zika ratio**	1.48 (1.33; 1.63)	0.787 (0.713; 0.870)	2.27 (1.87; 2.76)	1.07 (0.893; 1.28)
**Dengue 4**	**Pre-Zika**	262(234; 294)	220 (197; 246)	104 (82.4; 131)	75.4 (58.0; 97.9)

	**Post-Zika**	257(233; 284)	145 (131; 159)	189 (159; 224)	80.8 (64.9; 101)
	**Post-/pre-Zika ratio**	0.984 (0.894; 1.08)	0.659 (0.605; 0.718)	1.82 (1.51; 2.19)	1.06 (0.872; 1.29)

Dengue baseline serostatus was determined by $PRNT_{50}$ at M0; dengue seronegative was defined as titers < 10 (l/dil) against all four serotypes at baseline, and dengue seropositive, titers ≥ 10 (l/dil) against at least one dengue serotype at baseline.
Zika positive (Zika +ive) participants were those with Zika titers (microneutralization assay) at M72 ≥100 1/dil; Zika negative (Zika –ive) participants were those with M72 titers <100 1/dil.

References:

1. Zanluca C, Melo VC, Mosimann AL, Santos GI, Santos CN, Luz K. First report of autochthonous transmission of Zika virus in Brazil. Mem Inst Oswaldo Cruz 2015; 110(4): 569-72

2. Campos GS, Bandeira AC, Sardi SI. Zika Virus Outbreak, Bahia, Brazil. Emerging Infectious Diseases. 2015;21(10):1885-1886

3. Pan American Health Organization/World Health Organization. Timeline of the emergence of Zika virus in the Americas. Washington, DC: PAHO/WHO; 2016 Pan American Health Organization - wwwpahoorg - © PAHO/WHO, 2016. Last accessed November 2019.

4. World Health Organization. Zika virus infection – Honduras. 2015. https://www.who.int/csr/don/21-december-2015-zika-honduras/en/. Last accessed January 2020.
